# Work-to-family effects of inclusive leadership: The roles of work-to-family positive spillover and complementary values

**DOI:** 10.3389/fpsyg.2022.1004297

**Published:** 2022-11-08

**Authors:** Hong Zhu, Amy Y. Y. Chen

**Affiliations:** ^1^School of Tourism Management, Sun Yat-sen University, Zhuhai, China; ^2^School of Business, Hong Kong Baptist University, Kowloon, Hong Kong SAR, China

**Keywords:** inclusive leadership, work-to-family positive spillover, complementary values, family performance, work–family enrichment theory

## Abstract

Drawing on work–family enrichment theory, we explore whether inclusive leadership leads to employees’ work-to-family positive spillover, which further improves their family performance. We also focus on the moderating role of complementary values. A time-lagged study was conducted and the sample included 292 employees from two hotels. The results indicate that inclusive leadership triggers employees’ work-to-family positive spillover, and then their family performance is enhanced. Moreover, employees’ complementary values may strengthen the positive effect of inclusive leadership. We also provide theoretical and practical implications of the results.

## Introduction

Inclusive leadership, defined as “leaders who exhibit openness, accessibility, and availability in their interactions with followers” (Carmeli et al., 2010, p. 250), has drawn increasing attention in recent years. Researchers have found that inclusive leadership is positively related to subordinates’ affective organizational commitment and work engagement (Choi et al., 2015), psychological safety (Hirak et al., 2012; Javed et al., 2017; Wang and Shi, 2021), well-being (Choi et al., 2017), creativity (Carmeli et al., 2010; Javed et al., 2017, 2018), organizational citizenship behavior (Tran and Choi, 2019), and voicing behaviors (Yin, 2013; Jolly and Lee, 2021).

Despite the above research findings, studies on inclusive leadership are still in the early stage and more research attention is needed (e.g., Choi et al., 2017; Tran and Choi, 2019; Jolly and Lee, 2021; Wang and Shi, 2021). The current literature is mainly restricted to the influence of inclusive leadership in the work domain, leaving consequences in the family domain ignored. This omission is unfortunate, because family is the most important non-work domain and has significant impact on employees, including their work behaviors and well-being (Ford et al., 2007; Liu et al., 2013; Zhu et al., 2019; Ye et al., 2021). Moreover, owing to the increase of dual-career partners in the workforce, work role ambiguity/overload, and the blur of gender roles, organizations are presented with the challenge of improving employees’ work–family balance, and researchers are called for to pay more attention to work–family interface (Greenhaus and Allen, 2011; Michel et al., 2011; Liao et al., 2015; Cui and Li, 2021).

On the other hand, leaders are suggested to play a critical role in both employees’ work and family domains (e.g., Litano et al., 2016; Li et al., 2017; Zhang et al., 2019). However, current research on the impact of leadership on employees’ family life is still insufficient. A few studies focused on servant leadership (Zhang et al., 2012; Tang et al., 2016), ethical leadership (Liao et al., 2015; Zhang and Tu, 2018), authentic leadership (Zhou et al., 2019), and leader–member exchange (Liao et al., 2016), leaving inclusive leadership and other leadership styles under-examined. Researchers have thus also called for more studies to explore whether other leadership variables would exert effects on followers’ family performance (Liao et al., 2015; Zhang and Tu, 2018).

Responding to these appeals, this study focuses on the relationship between inclusive leadership and followers’ family performance, which indicates the degree to which individuals fulfill general responsibilities associated with the family (Carlson et al., 2010; Liao et al., 2016). Specifically, applying work–family enrichment theory, we further examine whether inclusive leadership leads to employees’ work-to-family positive spillover (WFPS), which might enhance their family performance in turn. Work–family enrichment theory suggests that resources gained from the work domain can be transferred to the family domain and therefore help employees enrich their family lives by meeting the requirements and expectations better in their families (Greenhaus and Powell, 2006; Tang et al., 2016). Inclusive leaders are open, accessible, and available to their subordinates; they usually initiate open communication to invite input from followers (Hollander, 2009; Wang and Shi, 2021). It is possible that employees are likely to transfer the initiation of open communication to their families, and show concerns for family members’ thoughts and interests. Accordingly, subordinates can generate WFPS, which refers to the process whereby positive moods and energy from work facilitate individuals’ roles in the family sphere (Grzywacz and Marks, 2000; Mennino et al., 2005). With the generation of WFPS, employees might benefit from the positive affect, skills, behaviors, and values transferred from the work domain, and thus their family performance can be improved resultantly. Therefore, this research aims to investigate whether inclusive leadership triggers employees’ WFPS, which further improves their family performance.

In addition, this research also sheds light on the boundary condition under which the impact of inclusive leadership can be strengthened or weakened. As suggested by the contingency perspective of leadership, the impact of leadership should be examined in consideration of the context in which it exits (Howell and Dorfman, 1981; Yukl, 2006). However, insufficient research has paid attention to the contextual factors that tune the impacts inclusive leaders exert on followers, with only two exceptions that work unit performance (Hirak et al., 2012) and leader-member exchange (Wang and Shi, 2021) were found to moderate the relationship between inclusive leadership and employee psychological safety. In this research, we focus on the moderating role of complementary values. Complementary values depict the degree to which the work values of an individual’s organization aligns with that of his/her family and community values (Duffy et al., 2017). When the level of complementary values is high, the work values are highly consistent with those of their families and communities, thus the affect, skills, behaviors, and values from employees’ work domain might be transferred to the family sphere more easily and smoothly. As a result, the positive impact of inclusive leadership on WFPS might be further enhanced. Hence, this research also aims to provide a comprehensive framework for understanding how inclusive leadership affects followers’ WFPS by examining the moderating role of complementary values.

To examine the above hypotheses, we conducted a questionnaire survey in two hotels located in Northern China. We choose this sample for two reasons. On the one hand, the service industry is increasingly vital in both developed and emerging countries (Liu et al., 2016). Taking China for an example, over 46.3% of the total employed population work in the service industry (Editoral Board of the China Commerce Yearbook, 2019). On the other hand, the service industry is characterized by long and irregular working hours, excessive workload, and difficulty in work-life balance (Lawson et al., 2013; Lee et al., 2016). In addition, Chinese people are suggested to possess high levels of familyism and place great emphasis on family life (Au and Kwan, 2009). Thus it is especially meaningful to conduct research on the work-family interface with a sample of hotels in China, which might facilitate organizations in the service industry tackle the challenges of helping employees balance work and family life (Zhang et al., 2019; Jolly and Lee, 2021).

**Figure 1:**
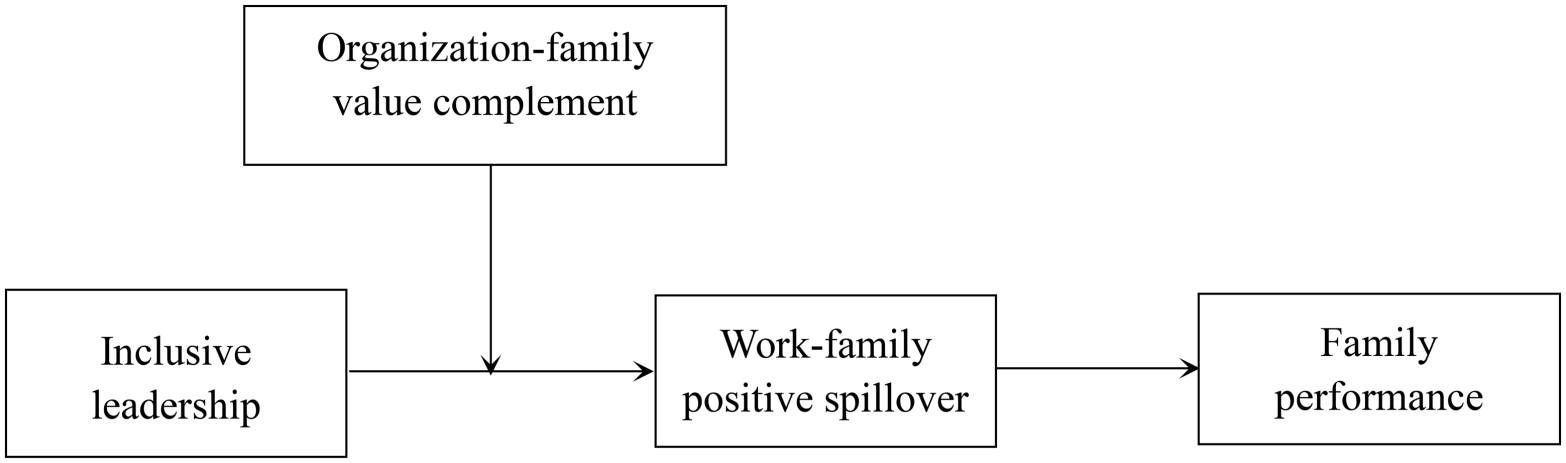
Conceptual model of this research.

The present research intends to contribute to the literature in several ways. First, it responds to the appeal for more attention to the topic of inclusive leadership and extends its consequences to the family domain for the first time. Specifically, to the best of our knowledge, we are among the first to examine the relationship between inclusive leadership and followers’ family performance. Second, the present study explores whether inclusive leadership triggers employees’ WFPS, which further affects their family performance. The examination of the linkage between inclusive leadership and WFPS is meaningful. In addition, the investigation of WFPS as the mediator provides a new theoretical perspective to understanding the process of inclusive leadership. Third, this research addresses a new moderator, i.e., complementary values, to help us have a better understanding of the boundary conditions of inclusive leadership. The theoretical model for this study is shown in Figure 1.

## Theory and hypotheses development

### Inclusive leadership

Nembhard and Edmondson (2006) were among the first to coin the concept of leader inclusiveness, which refers to leader behaviors that invite and appreciate inputs from subordinates, leading to the beliefs that “their voices are genuinely valued” (p. 948). Hollander (2009) further suggested inclusive leadership as a type of relational leadership in which the core is cultivating high quality relationships with followers by paying attention to their needs and interests and being available to them. Afterwards, Carmeli et al. (2010) developed the construct of inclusive leadership and defined it as “leaders who exhibit openness, accessibility, and availability in their interactions with followers” (p. 250).

In the present research, we adopt the definition from Carmeli et al. (2010), which comprises of three dimensions, i.e., openness, availability, and accessibility. Specifically, openness indicates the degree to which leaders demonstrate openness by deeds such as inviting followers to contribute in decision making, valuing followers’ different opinions and perspectives, and facilitating the generation of new ideas and methods to solve problems (Carmeli et al., 2010; Hirak et al., 2012; Tran and Choi, 2019). Availability refers to the degree that followers perceive their leaders as available to them both physically and psychologically, and are willing to provide timely assistance to them whenever they encounter difficulties and problems (Nembhard and Edmondson, 2006; Hirak et al., 2012; Choi et al., 2017). Accessibility means that a leader builds a close relationship with his or her followers by exhibiting behaviors such as acknowledging followers’ contributions, sharing visions with them, and concerning about their expectations, interests and feelings (Carmeli et al., 2010; Choi et al., 2015).

As we have discussed before, the construct of inclusive leadership has drawn increasing attention recently, providing evidence that it can have notable impacts on followers’ attitudes, behaviors and psychological well-being (e.g., Choi et al., 2015; Javed et al., 2018; Tran and Choi, 2019). However, the research on inclusive leadership is still in its infancy and more empirical evidence is needed (Tran and Choi, 2019; Jolly and Lee, 2021; Wang and Shi, 2021). Thus, we respond to the call by extending the consequences of inclusive leadership to the family domain. Especially, we focus on the effects of inclusive leadership on followers’ family performance by examining the mediating role of WFPS and the moderating role of complementary values.

### Inclusive leadership, WFPS, and family performance

WFPS is defined as “the transfer of positively valenced affect, skills, behaviors, and values from the work domain to the family domain, thus having beneficial effects on the family domain” (Hanson et al., 2006, p. 251). For instance, the positive affect created in the workplace can be transferred in the family, the values and skills gained in the work domain can be applied in the family, and the behaviors one learned in the workplace can also be initiated in the family life (Hanson et al., 2006; Masuda et al., 2012; Tang et al., 2016). WFPS is distinct from work-family enrichment (WFE), which is also a construct focusing on positive work–family interface (Carlson et al., 2006; Hanson et al., 2006). WFPS occurs when employees transfer the gains from the work domain to the family domain, while WFE occurs when the gains transferred from work result in a higher quality of life at home (Wayne, 2009; Masuda et al., 2012). In this research, we hypothesize that the benefits (e.g., positive affect, values, skills, and behaviors) generated by inclusive leadership can be transferred to followers’ family domain, leading to the generation of WFPS, which further improve their family performance.

According to work-family enrichment theory, the positive impact of leadership on employees’ family life can be exerted through two paths, i.e., the instrumental path and the affective route (Greenhaus and Powell, 2006; Zhang and Tu, 2018). Herein we suggest that WFPS from inclusive leadership is generated through these two paths. *Via* the instrumental path, the perspectives, values, and knowledge that employees learned from their inclusive leaders can be transferred into their family domain, leading to the generation of WFPS. As discussed before, inclusive leaders exhibit openness, availability, and accessibility towards followers, and initiate behaviors to make them feel being valued (Carmeli et al., 2010; Randel et al., 2018; Tran and Choi, 2019). Employees observe their inclusive leaders’ behaviors in the workplace, and perceive the favor from inclusive leaders. When employees return back home, they are possible to recall and imitate inclusive leaders’ behaviors in the family life. Followers might thus initiate openness, availability, and accessibility towards family members.

As for the second path, i.e., the affective route, inclusive leadership could promote the positive affect within the followers (Choi et al., 2015; Tran and Choi, 2019), which, in turn, generates their positive affect in the family domain, leading to the generation of WFPS. As suggested by work-family enrichment theory, the positive affect produced by psychological resources (e.g., psychological safety and self-esteem) at work is associated with an outward focus of attention, which further leads to warm and caring interactions at home (Greenhaus and Powell, 2006). To the extent that inclusive leaders’ behaviors often signal benevolence by showing genuine concern and caring to employees (Burke et al., 2007), they tend to perceive sense of belonging and being valued (Randel et al., 2018). Thus employees’ experiences with inclusive leaders are usually positive (e.g., Yin, 2013; Tran and Choi, 2019). These experiences improve employees’ affective states (Ramamoorthy et al., 2005; Choi et al., 2017), which can help them accommodate family roles better (Carlson et al., 2006). As such, employees might extend the positive feelings generated from inclusive leaders to their family life, leading to positive emotional states at home and caring interaction towards family members. Thus, WFPS is produced *via* the affective route as well.

To summarize, the positive spillover effects of inclusive leadership to followers’ family life can be generated from both the instrumental path and the affective route. The instrumental benefits of behavior-base resources (e.g., values and habits) and affective benefits of increased positive emotional states (e.g., positive feelings and psychological well-being) generated from inclusive leadership can both be transferred from the work domain to the family domain. Hence, we hypothesize the following:

*Hypothesis 1*: Inclusive leadership is positively related to WFPS.

Work–family enrichment theory asserts that resources acquired at work can be transferred to the family domain and thus help employees meet their families’ requirements and expectations and enrich their family lives (Greenhaus and Powell, 2006; Wayne, 2009). It demonstrates the important role of psychological and skill resources in understanding how and why work experiences can enhance employees’ personal lives (Greenhaus and Powell, 2006; Masuda et al., 2012).

WFPS indicates the successful transfer of valuable affect, skills and behaviors from the work domain to the family domain (Hanson et al., 2006). According to work–family enrichment theory, WFPS can facilitate individuals dealing with personal and practical issues at home, thus their family performance can be improved (Greenhaus and Powell, 2006). That is, with the generation of WFPS from inclusive leadership, employees’ family performance might be enhanced by stimulated motivation, improved ability and skills, and persistence, etc. (Greenhaus and Powell, 2006; Liao et al., 2015).

Specially, on the one hand, with the ability and skills achieved from WFPS (Wayne, 2009), employees might have a good understanding of family members’ needs and expectations, and they are capable of fulfilling family requirements and responsibilities better. On the other hand, with higher WFPS, employees usually have a good mood (Hammer et al., 2005), thereby they are likely to devote more persistence to meet the needs of family members and complete family tasks, hence followers’ family performance is enhanced. Similarly, previous research has suggested that a positive mood can improve individuals’ performance and rewards by enhancing their cognitive functioning, task and interpersonal activity and persistence (Edwards and Rothbard, 2000; Greenhaus and Powell, 2006).

Based on the above arguments, we hypothesize that:

*Hypothesis 2*: WFPS is positively related to employees’ family performance.

We have discussed that followers tend to gain a series of psychological resources from inclusive leadership and transfer the positive values and perspectives they have experienced at work to home. These resources from work could spill over to followers’ family domain and contribute to their family life, producing WFPS (Hanson et al., 2006). As a result, the followers’ family performance can be improved. Taken together, inclusive leadership is positively related to followers’ WFPS, which in turn enhances their family performance. Hence we hypothesize that:

*Hypothesis 3*: WFPS mediates the relationship between inclusive leadership and employees’ family performance.

### The moderating effect of complementary values

As suggested by the contingency theory of leadership, the extent to which leadership influences followers is heavily dependent on the context where it occurs (Howell and Dorfman, 1981; Yukl, 2006). A potential context of inclusive leadership is the alignment between organizational values and family values. Responding to the call of examination on the boundary role of work-family value alignment (Li et al., 2017), we suggest that complementary values might play a moderating role in the relationship between inclusive leadership and WFPS. Complementary values, or organizational values that complement family and social values (Duffy et al., 2017), indicate the degree to which the work values of an individual’s organization aligns with his/her family and community values (Duffy et al., 2017). When the degree of complementary values is high, employees perceive a high correspondence between values of the organization and those adopted in their families (Duffy et al., 2017). If the level of complementary values is low, employees might find that things valued in the organizations are not appreciated in their family or community (Duffy et al., 2017).

To the extent that the values employees perceive at work are complementary with the values in their family, they are prone to extend the values from work to family. As leaders are usually considered as representatives of organizations, their behaviors are deemed to be reflections of the organizational values (Lord and Brown, 2001; Driscoll and McKee, 2007). Accordingly, when employees perceive the values of “appreciating the uniqueness of individuals” from inclusive leaders in the organization (Holvino et al., 2004; Randel et al., 2018), and they believe that such value is in consistence with those in their family, they are more willing to transfer the values they have learned from leaders to their family domain, leading to a higher level of WFPS. On the contrary, if the level of complementary values is low, employees might find that things valued in the organizations are not appreciated in their family (Duffy et al., 2017). In this situation, those positive experiences from work are less likely to be extended to their family, suppressing the enhancement of WFPS. Based on the above arguments, we hypothesize the following:

*Hypothesis 4*: Complementary values moderate the relationship between inclusive leadership and WFPS such that the higher the level of complementary values, the stronger the relationship between inclusive leadership and WFPS.

## Materials and methods

### Sample and procedures

We conducted this study in two hotels in northern China. The human resource departments of these two hotels aided in the data collection process. The human resource managers introduced the purpose of the study and the procedures arranged to collect data. The voluntary and anonymous nature of the participation was also highlighted. Then each participant was distributed a questionnaire and a return envelope. Participants could return the sealed questionnaire to a box in the human resource department.

To reduce the risk of common method bias (Podsakoff et al., 2003), we have designed a time-lagged survey at three time points to collect the data. Each participant was coded to match their responses at three time waves. In the first wave of survey, we randomly selected 600 frontline employees from the name lists provided by the hotel’s human resource departments. These employees were required to report their demographic information (gender, age, education, and job tenure with immediate supervisor) and their perception of inclusive leadership. In the second wave of survey, which was conducted 2 months later, we invited these employees to participate in this study again and rate their WFPS and complementary values. After another 2 months, in the third wave of survey, the employees rated their family performance.

At Time 1, 453 usable questionnaires were received, generating a response rate of 75.50%. At Time 2, we gathered 382 completed questionnaires (84.32% response rate). In the final wave, 292 completed questionnaires were received, with a response rate of 76.44%. Accordingly, the final sample consisted 292 employees. The demographic information of these employees is summarized as follows. The employees’ average age was 35.65 years old (SD = 10.56), and the average job tenure with their immediate supervisor was 2.65 years (SD = 2.09). Among the participants, 83.11% held a degree of high school or below, and 51.35% of them were female.

### Measures

#### Inclusive leadership

We assessed inclusive leadership with the 9-item measure from Carmeli et al. (2010). Each item used a 5-point Likert scale. The response options of the measure ranged from 1, “strongly disagree,” to 5, “strongly agree.” “My manager is an ongoing ‘presence’ in this team—someone who is readily available.” is a sample item. The second-order factor model indicated a good fit (χ^2^(24) = 83.38, CFI = 0.97, TLI = 0.95, RMSEA = 0.09). The Cronbach’s alphas were 0.88, 0.86, and 0.88 for the three dimensions, respectively, and 0.93 for the construct.

#### Work-to-family positive spillover

Work-to-family positive spillover was measured by the 11-item scale developed by Hanson et al. (2006). Each item used a 5-point Likert scale. The response options of the measure ranged from 1, “strongly disagree,” to 5, “strongly agree.” Sample items are “When things are going well at work, my outlook regarding my family life is improved.,” and “Being in a positive mood at work helps me to be in a positive mood at home.” The second-order factor model indicated a good fit (χ^2^(41) = 159.29, CFI = 0.95, TLI = 0.94, RMSEA = 0.09). The Cronbach’s alphas were 85, 0.92, and 0.87 for the three dimensions, respectively, and 0.94 for the construct.

#### Complementary values

We evaluated complementary values by the scale developed by Duffy et al. (2017). Each item used a 5-point Likert scale. The response options of the measure ranged from 1, “strongly disagree,” to 5, “strongly agree.” “The values of my organization match my family values.” is a sample item. The Cronbach’s alpha of this construct was 0.90.

#### Family performance

Family performance was evaluated with the 5-item measure developed by Carlson et al. (2010). Each item used a 5-point Likert scale. The response options of the measure ranged from 1, “strongly disagree,” to 5, “strongly agree.” A sample item is “I can fulfill all the family responsibilities.” The Cronbach’s alpha of this construct was 0.87.

#### Control variables

We have included employees’ demographic information, including age, gender, job tenure with the immediate supervisor, education level, and number of kids as control variables. As these variables have been suggested to affect employee family performance (Liao et al., 2016; Zhou et al., 2019), we intended to exclude their potential impacts on our results by controlling them. Moreover, we also created two dummy variables to control the differences between the two hotels we surveyed.

## Results

### Construct validity of measurement

Utilizing AMOS 17.0, confirmatory factor analyses were conducted to assess the discriminant and convergent validity of the core constructs in our theoretical model. Table 1 presents results of the confirmatory factor analyses, demonstrating that the four-factor model generated a good fit (*χ*^2^(59) = 78.96, TLI = 0.98, CFI = 0.99, and RMSEA = 0.03). Thus the factors’ discriminant validity was supported. Moreover, the factor loadings of all of the items in the four-factor model were significant (greater than 0.55), supporting the convergent validity of the four constructs.

**Table 1:** Confirmatory factor analysis.

Model	*χ* ^2^	*df*	TLI	CFI	RMR	RMSEA
Four factors (baseline model)	78.96	59	0.98	0.99	0.03	0.03
Three factors (combine inclusive leadership and organization-family value complement)	593.66	62	0.70	0.76	0.12	0.17
Three factors (combine work-family positive spillover and family performance)	472.94	62	0.76	0.81	0.06	0.15
One factor (combine all items into one factor)	1367.59	66	0.30	0.41	0.15	0.26

*N* = 292. CFI = comparative fit index, TLI = Tucker-Lewis Index, RMSEA = root mean square error of approximation.

All variables were packaged into three parcels.

### Descriptive statistics and correlations

Table 2 shows the means, standard deviations, and zero-order Pearson correlations for all key variables in this study. As presented in Table 2, inclusive leadership had a positive correlation with WFPS (*r* = 0.22, *p* < 0.01), and WFPS had a positive correlation with employee family performance (*r* = 0.51, *p* < 0.01). Thus our hypotheses were initially supported.

**Table 2:** Means, standard deviations, correlations, and reliabilities.

Variables	Means	SD	1	2	3	4	5	6	7	8	9	10	11
1. Employee gender	1.51	0.50	1										
2. Employee age	35.64	10.54	−0.09	1									
3. Employee education	1.20	0.49	−0.09	−0.16^**^	1								
4. Employee tenure	2.65	2.11	−0.21^**^	0.12^*^	0.11	1							
5. Number of kids	1.1	0.95	0.13^*.^	0.13^*^	−0.10	0.13^*^	1						
6. Hotel 1	0.58	0.49	−0.02	−0.06	−0.05	0.23^**^	0.10	1					
7. Hotel 2	0.42	0.49	0.02	0.06	0.05	−0.23^**^	−0.10	−1.00^**^	1				
8. Inclusive leadership	4.03	0.82	−0.14^*^	0.11	−0.01	−0.01	0.07	−0.06	0.06	1			
9. Organization-family value complement	3.42	0.88	0.06	0.04	0.09	−0.05	0.09	0.08	−0.08	0.21^**^	1		
10. Work-family positive spillover	4.01	0.69	−0.03	0.20^**^	−0.04	0.00	0.13^*^	−0.15^*^	0.15^*^	0.22^**^	0.17^**^	1	
11. Family performance	3.56	0.75	0.03	0.06	−0.03	−0.15^*^	0.09	−0.01	0.01	0.18^**^	0.27^**^	0.51^**^	1

*N* = 292. Gender was coded as “1” for male and “2” for female; Education was coded as “1” for high school degree or below, “2” for junior college degree, “3” for bachelor degree, and “4” for graduate degree or higher;

* *p* < 0.05 and

** *p* < 0.01 (two-tailed).

### Hypotheses testing

As shown in Table 3, with employee demographics and hotel differences as control variables, inclusive leadership had a positive effect on WFPS (*β* = 0.19, *p* < 0.01, Model 2), supporting Hypothesis 1. Moreover, WFPS had a significant positive impact on employee family performance (*β* = 0.51, *p* < 0.01, Model 7), supporting Hypothesis 2.

**Table 3:** Hierarchal analysis results.

	Work-family positive spillover				Family performance			
	M1	M2	M3	M4	M5	M6	M7	M8
*Control variables*								
Employee gender	−0.03	−0.01	−0.02	−0.01	−0.00	0.02	0.01	0.02
Employee age	0.17^**^	0.15^*^	0.15^*^	0.15^*^	0.07	0.05	−0.02	−0.03
Employee education	−0.01	−0.01	−0.03	−0.04	0.02	0.02	0.02	0.02
Employee tenure	−0.02	−0.01	0.01	0.01	−0.20^**^	−0.18^**^	−0.19^**^	−0.18^**^
Number of kids	0.12^*^	0.11^*^	0.09	0.07	0.11	0.09	0.04	0.04
Hotel 1	−0.13^*^	−0.13^*^	−0.14^*^	−0.14^*^	−0.04	0.04	0.10	0.11
*Independent variable*								
Inclusive leadership		0.19^**^	0.16^**^	0.20^**^		0.17^**^		0.07
*Mediator*								
Work-family positive spillover							0.51^**^	0.51^**^
*Moderator*								
Organization-family value complement			0.14^*^	0.12				
*Interaction*								
Inclusive leadership × organization-family value complement				0.13^*^				
*R* ^2^	0.07	0.10	0.12	0.13	0.04	0.07	0.29	0.30
Δ*R*^2^	0.07	0.03	0.02	0.01	0.04	0.03	0.25	0.23
*F*	3.10^**^	4.12^**^	4.32^**^	4.34^**^	1.96	2.75^**^	15.03^**^	13.58^**^
Δ*F*	3.10^**^	9.60^**^	5.28^*^	4.07^*^	1.96	7.21^**^	89.17^**^	83.15^**^

*N* = 292.

* *p* < 0.05 and

** *p* < 0.01 (two-tailed).

Hypothesis 3 predicted that WFPS mediates the relationship between inclusive leadership and followers’ family performance. In support of Hypothesis 3, the results indicated that WFPS had a significant positive impact on employee family performance (*β* = 0.51, *p* < 0.01, Model 8), whereas the influence of inclusive leadership on family performance was not significant (*β* = 0.07, n.s., Model 8). We calculated the confidence interval of the indirect influence of inclusive leadership on employee family performance through WFPS. The result indicated a significantly positive indirect effect (estimate = 0.07, S.E. = 0.09, 95% CI = [0.032, 0.153]). Hence, Hypothesis 3 was supported.

**Figure 2:**
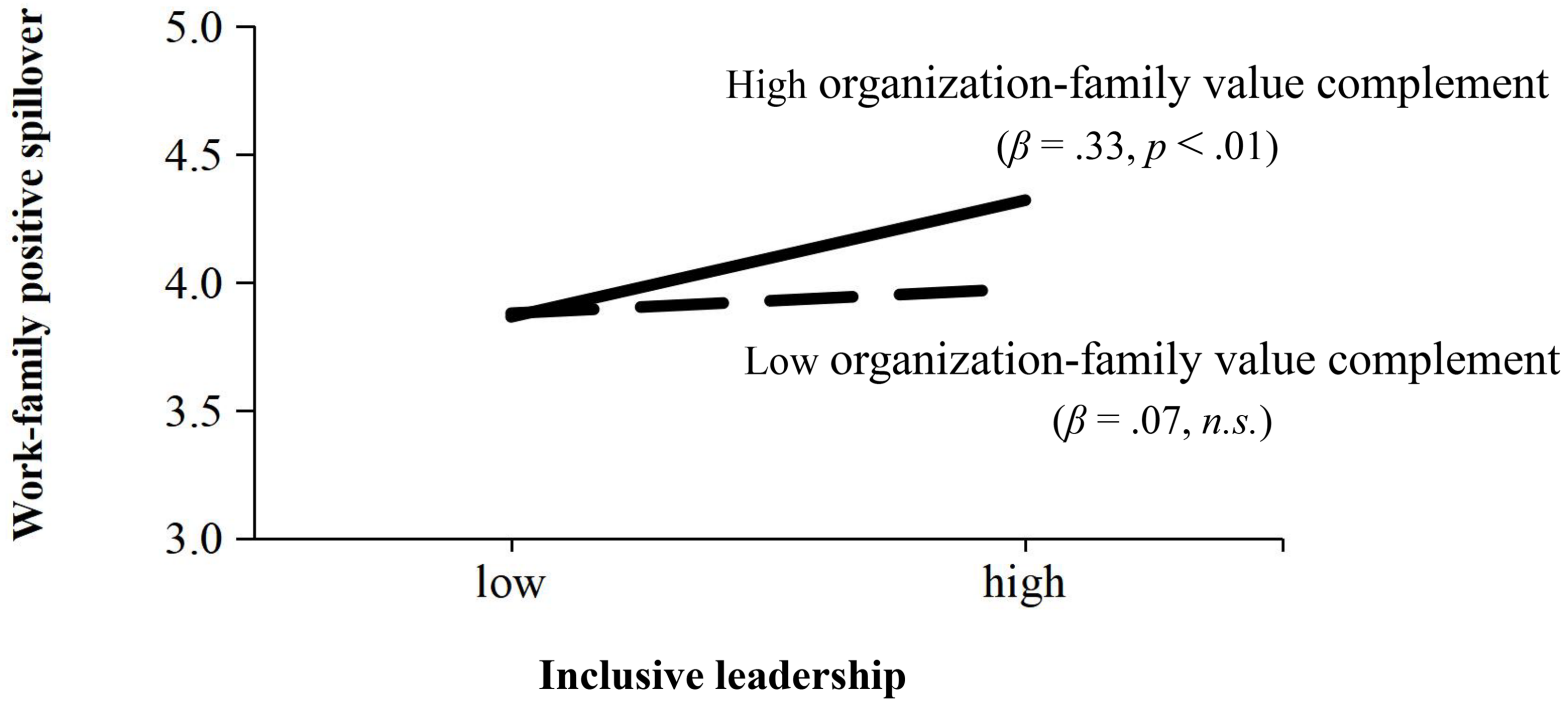
Interaction of inclusive leadership and work-family value complement on work-family positive spillover.

To examine Hypothesis 4, an interaction between inclusive leadership and complementary values was generated with standardized values (Aiken et al., 1991). The path analysis results (Table 3) showed that the interaction between inclusive leadership and complementary values had a positive relationship with WFPS (*β* = 0.13, *p* < 0.01, Model 4), supporting Hypothesis 4. To have a better understanding of the moderating effect, we plotted the interaction following Aiken et al.’s (1991) procedures. As presented in Figure 2, the positive influence of inclusive leadership on WFPS was significant for employees with a high level of complementary values (*β* = 0.33, *p* < 0.01) but nonsignificant for employees with low complementary values (*β* = 0.07, n.s.). Therefore, Hypothesis 4 was supported further.

## Discussion

By conducting a time-lagged study, we investigated when and why inclusive leadership improves followers’ family performance. Applying work-family enrichment theory, we tested that inclusive leadership triggered employees’ work-to-family positive spillover, which further improved their family performance. Moreover, we also found that complementary values reinforced the positive impact of inclusive leadership on WFPS.

### Theoretical implications

This study contributes to the literature in the following ways. First, it advances the literature on inclusive leadership by extending its consequences into followers’ family life. As the research on inclusive leadership is still in its infancy (e.g., Carmeli et al., 2010; Choi et al., 2017), there has been scant attention toward how inclusive leadership affect followers’ family life. Previous research is mainly restricted to work outcomes, such as creativity (Carmeli et al., 2010; Choi et al., 2015; Javed et al., 2017) and organizational citizenship behavior (Tran and Choi, 2019). Hence our study helps fill this research gap in the area of inclusive leadership. Moreover, although leaders are suggested to “play a particularly important role in helping individuals balance their work and family demands” (Kailasapathy et al., 2014, p. 2682), the focus of leadership research mainly remains on followers’ work domain, leaving the cross-domain effects of leadership on followers’ family life understudied (Li et al., 2017; Zhang and Tu, 2018). Thus this research also contributes to the leadership literature by shifting concerns from the work domain to followers’ family domain.

Second, this study depicts the influencing mechanism of inclusive leadership on employees’ family performance by adopting the work–family enrichment theory. Previous research has mainly explained the impact of inclusive leadership on followers from the social exchange perspective (e.g., Choi et al., 2015; Javed et al., 2017, 2018; Tran and Choi, 2019). This research departs from the previous literature of inclusive leadership by applying a new theoretical perspective, i.e., the work-family enrichment theory. Drawing on this theory, we find that inclusive leadership is positively related to followers’ WFPS, which further leads to their improved family performance. This finding reveals a new mediating mechanism that transfers the positive impact of inclusive leadership on followers’ family life. To the best of our knowledge, this research is among the first to investigate WFPS as a critical intervening mechanism that underlies the relationship between inclusive leadership and family outcomes.

Third, this research investigated the moderating role of complementary values, which adds more empirical evidence on the boundary conditions that influence the degree to which inclusive leaders impact followers. Given the fact that current research findings about the boundary conditions under which inclusive leaders can exert effects on followers are still limited and insufficient (Wang and Shi, 2021), it is meaningful to examine a new moderator, i.e., complementary values. The identification of this new moderator enriches our understanding about how inclusive leaders can exacerbate its positive effects on followers, responding to the call for more examination of comprehensive moderators in the research of inclusive leadership (Wang and Shi, 2021).

### Practical implications

The research findings from this study can delineate notable implications for organizations. Given the fact that family is the most important non-work domain for employees, and family life impacts work-related outcomes significantly (Liu et al., 2013; Xin et al., 2018), it is important to improve employees’ family lives. Our research findings suggest that inclusive leaders lead to followers’ WFPS, which further benefits their family performance. Therefore it is urgent for organizations to encourage leaders to adopt the inclusive model of behaviors. Organizations might provide training programs for leaders, and encourage them to be open, available, and accessible to followers (Carmeli et al., 2010). For instance, leaders can be trained to initiate behaviors including inviting followers to participate in decision making, providing timely assistance to them when they have difficulties, building close relationships with them, and showing concerns about followers’ expectations and feelings (Carmeli et al., 2010; Hirak et al., 2012; Choi et al., 2015; Tran and Choi, 2019).

Moreover, as shown by our research findings, complementary values strengthen the positive linkage between inclusive leadership and WFPS. Therefore, organizations should pay more attention to the complementary values, i.e., the high alignment between values adopted in the organization and employees’ families. With the existence of complementary values, the positive effects of inclusive leadership can be transferred into WFPS more easily. Organizations can take measures to enhance the level of employees’ complementary values. For instance, training programs can be provided to enhance employees’ understanding about core values adopted in the organization, and further improve the chance of alignment between work values and family/community values.

### Limitations and future directions

Notwithstanding the importance of its findings, this research has several limitations. First, we collected data at two time points. Specially, the data about inclusive leadership and complementary values were collected at Time 1, while that about WFPS and family performance were at Time 2 and Time 3, respectively. However, the study might still potentially be susceptible to common method bias. Moreover, due to resource constraints, the data of family performance was self-reported by followers rather than their family members. Although some of the previous research on family performance also adopted the same data source (e.g., Liu et al., 2016; Li et al., 2021), it might still cause concerns. For instance, as family performance rated by followers themselves might be higher than that rated by their family members, the impact of inclusive leadership *via* WFPS might be inflated. Moreover, due to the limitation of survey research, we cannot confirm the causal relationship in this study. Therefore, we encourage future research to apply other methods, such as collecting data about family performance from followers’ family members and using experiments to improve the research design.

Moreover, this study was conducted in China, where individuals usually attach high importance to families and the level of family involvement is high (Greenhaus and Powell, 2006; Au and Kwan, 2009). With this characteristic, Chinese people might make a better use of work-generated resources in their family lives, and thus benefiting more from these emotional and instrumental resources generated in the workplace than their western counterparts (Greenhaus and Powell, 2006; Tang et al., 2016). As a result, the issue of generalizability of our research findings is raised. Cross-cultural research is needed to validate whether inclusive leadership can facilitate followers’ family performance *via* the generation of WFPS in non-Chinese contexts.

In addition, our research examined an important perspective for understanding how inclusive leadership impacts followers’ family performance by illuminating the role of WFPS, but we do not necessarily rule out the possibility of other mediating mechanisms. Future research could also extend our work and explore other mechanisms that underlie the relationship between inclusive leadership and followers’ family outcomes. For instance, future research could develop a measurement of inclusive behavior at home, and examine whether it play a mediating role in the relationship between inclusive leadership and followers’ family outcomes. Similarly, Liao et al. (2016) have suggested that ethical leadership mediated the relationship between ethical leadership at the workplace and life satisfaction.

Moreover, as there is little research focusing on the antecedents of inclusive leadership, it is urgent for researchers to examine organizational factor and individual characteristics that might spur the emergence of inclusive leadership.

## Conclusion

Drawing on work-family enrichment theory, this research demonstrates that inclusive leadership leads to employees’ increased WFPS, which further improves their family performance. Moreover, the direct impact of inclusive leadership on WFPS can be attenuated by followers’ complementary values. These research findings provide solid evidence for the positive effects of inclusive leadership and urge future research to focus on this filed more and investigate its pivotal impact.

## Data availability statement

The raw data supporting the conclusions of this article will be made available by the authors, without undue reservation.

## Author contributions

HZ was in charge of designing the theoretical model, collecting data, and writing the manuscript. AC processed the data and revised the manuscript. All authors contributed to the article and approved the submitted version.

## Funding

We thank the support provided by National Natural Science Foundation of China (Grant No. 71702198).

## Conflict of interest

The authors declare that the research was conducted in the absence of any commercial or financial relationships that could be construed as a potential conflict of interest.

## Publisher’s note

All claims expressed in this article are solely those of the authors and do not necessarily represent those of their affiliated organizations, or those of the publisher, the editors and the reviewers. Any product that may be evaluated in this article, or claim that may be made by its manufacturer, is not guaranteed or endorsed by the publisher.
